# Plantar foot vibration thresholds: a comparison between measurements with seated and standing subjects

**DOI:** 10.1186/1757-1146-5-S1-O23

**Published:** 2012-04-10

**Authors:** AMC Germano, G Schlee, TL Milani

**Affiliations:** 1 Department of Human Locomotion, Chemnitz University of Technology, Chemnitz, Germany

## Background

The importance of the somatosensory information from the plantar foot area for balance control is well reported in the literature. Usually, tests for touch and vibration sensitivity are performed with subjects in sitting or supine position [1]. However, balance tests are measured in standing position [2]. Therefore the aim of this study was to compare vibration thresholds measured with subjects seated and during standing.

## Materials and methods

Sixty-six healthy subjects of both genders with a mean age of 22.1 (± 3.2) years participated in this study. Vibration perception thresholds [µm] were measured at 200Hz in two conditions: sitting (90 ° knee angle) and standing on both legs. Five measurements with increasing amplitude were performed at each of the three analyzed anatomical locations of the right plantar foot: heel, first metatarsal head (MET I) and hallux. The contact force between the vibration probe and the anatomical locations as well as foot temperature were controlled throughout the experiments. Body position and anatomical locations were randomized between the subjects.

## Results

**Figure 1:**
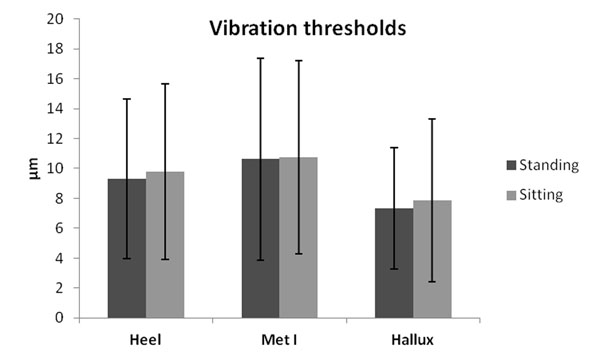
Vibration thresholds for the standing and sitting conditions.

The contact forces between the probe and the location were higher for standing in comparison to sitting condition (27, 7% hallux, 43, 2% Met I and 62, 6% Heel). However, no significant differences in vibration thresholds between standing and sitting conditions were found in any of the analysed anatomical locations (Figure 1).

## Conclusions

Cassella et al. (2000) demonstrated that greater contact forces between the probe and the location being analysed are reducing vibration thresholds. However, despite the higher force applied by the probe during standing, no significant differences between the tested positions were evaluated in the present study. It seems that the standing position affects the perception of vibration stimuli, since subjects need to concentrate on different tasks, e.g. keep their balance. This could explain the lack of differences between thresholds measured in the different positions, although contact forces increased during standing.
